# Supplementary material for: Flexibility of functional neuronal assemblies supports human memory
Source: Nat Commun. 2022 Oct 18;13:6162. doi: 10.1038/s41467-022-33587-0 (PMC9579146; doi:10.1038/s41467-022-33587-0)
Supplement: Supplementary file 1 — Supplementary Information [file 41467_2022_33587_MOESM1_ESM.pdf]

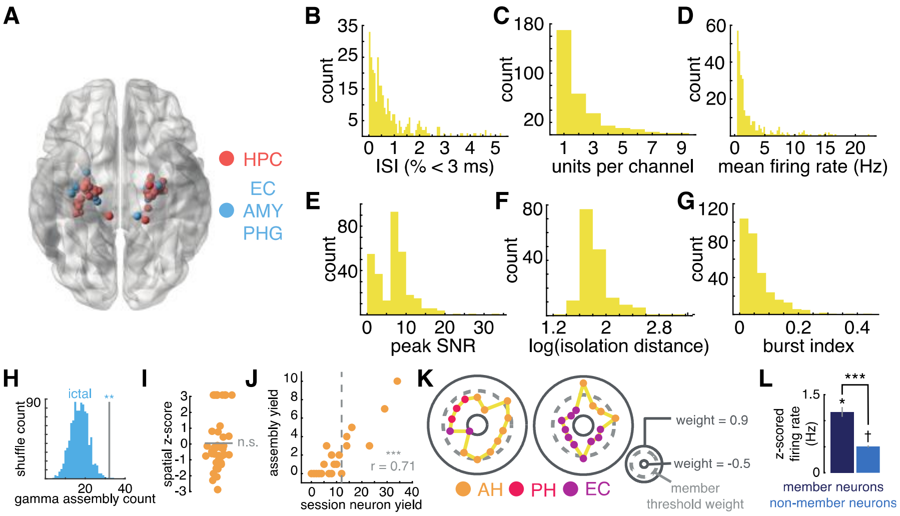

Supplementary Fig. 1. Electrode localizations, unit quality metrics, and assembly identification control analyses. (**A**) Localizations of electrodes used to isolate single units used in the study. (**B**) Percentage of inter-spike intervals (ISI) less than 3 ms across neurons. (**C**) Distribution of unit yield per channel. (**D**) Distribution of firing rates of neurons used for assembly identification. (**E**) Average ratio of peak unit spike voltage to signal noise across neurons. (**F**) Separability of waveforms isolated from the same microwire, for all relevant neurons. (**G**) Burst index of all neurons used in assembly identification. (**H**) Identification of a significant fraction of neuronal assemblies after removing leads demonstrating inter-ictal epileptiform discharges at some point during implantation (left, $p = 0.0030$). (**I**) Distribution of z-scores representing the anatomical clustering of assemblies (one-tailed test, $n = 45$, $p = 0.83$). When comparing spatial clustering by hemisphere, region, and channel separately, we noted a marginal but significant spatial clustering of assembly neurons by hemisphere (89% versus 78%, $\chi 2 = 9.4$, $p = 0.0023$), region (56% versus 39%, $\chi 2 = 14.6$, $p = 1.3e-4$), and channel (26% versus 13%, $\chi 2 = 18.0$, $p = 2.2e-5$). (**J**) Correlation between the number of single units and assemblies identified during a recording session (Spearman rank correlation, $n = 38$, $r = 0.71$, $p = 6.49e-7$). (**K**) Three additional examples of neuronal assemblies following the convention outlined in Fig. 1. (**L**) Mean (bar height) ± s.e.m (error bar) of the z-scored firing rates of all assembly member (dark blue) and non-member (blue) neurons demonstrating that the firing rate of members ($n = 124$) significantly exceeds that of non-members ($n = 770$) and of baseline (Wilcoxon rank sum; members to non-members, $p = 0.049$; members to baseline, $p = 0.068$; non-members to baseline, $p = 3.6e-20$). n.s. = not significant. † = $p < 0.10$. *$p < 0.05$. **$p < 0.01$. ***$p < 0.001$. Source data are provided as a source data file.

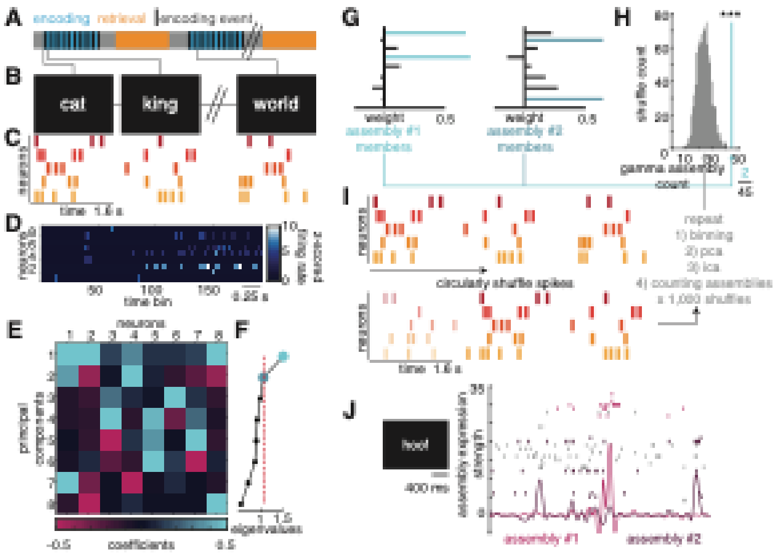

Supplementary Fig. 2. Assembly identification and statistical method. (**A**) Schematic of free recall task with interleaved encoding periods (blue) during which word stimuli appear on screen (black, (**B**)) and retrieval periods (orange) during which participants recall words from the previous encoding list. (**B**) Example of appearance of word stimuli on screen during encoding periods. (**C**) Sham data illustrating spiking data during encoding events. Spiking data from all encoding events across the recording session were used to identify assemblies. (**D**) Three seconds of binned firing rate data example session. Each bin is 25 ms in duration. Spikes from encoding events are segregated into non-overlapping bins, generating a firing rate for each bin. These firing rates are then normalized (z-scored) based on the distribution of firing rates observed from that session. (**E**) Principal component analysis (PCA) coefficient matrix. Each value in the matrix communicates the degree to which a given neuron (x-axis) influences a given principal component (y-axis) (**F**) Each principal component is associated with an eigenvalue. The Marchenko-Pastur law dictates the expected upper bound of PCA eigenvalues stemming from patterns with *independent* components. In other words, patterns with eigenvalues above this limit is due to co-dependence of the individual variables (neurons) contributing to the pattern. This is a functional definition for a cell assembly. (**G**) Assembly patterns identified in (**F**) are then optimized via independent component analysis (ICA). This assigns each neuron a "weight," the degree to which it contributes to the assembly. See methods for more information on why this step is included. (**H**) Distribution of assemblies identified after shuffling the individual session spike trains (gray histogram) (**I**) and repeating assembly identification 1,000 times compared to the actual number of assemblies identified (blue line). The illustrated assemblies in (**H**) are 2 of the 45 actual assemblies used in the analysis. (**I**) Illustration of the circular shuffle procedure done 1,000 times proximal to repeating assembly identification. (**J**) Example of spike assembly activity (assembly #1 and assembly #2) during an example encoding event (while "hoof" was displayed on screen for 1.6 s) with the spike raster superimposed (y-axis = neuron identity). The color of the assembly activity curve matches the color of the neurons comprising that assembly.

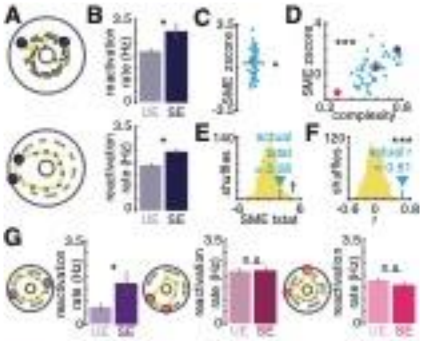

Supplementary Fig. 3. Activation of complex assemblies predicts successful memory encoding. (**A**) Two example assembly schematics with member neurons enlarged and colored. (**B**) Activation rates of the two assemblies displayed in (A) during unsuccessful (UE, light) and successful (SE, dark) encoding events (Wilcoxon two-group rank sum; top, $z\ (n = 21+144) = 2.256$, $p = 0.012$; bottom, $z\ (n = 75+225) = 3.050$, $p = 0.001$). Bar height represents the mean activation rate across events and errors bars the s.e.m. (**C**) Distribution of z-scores obtained by comparing the SE and UE activation rate distributions for each assembly (one-sample t-test versus zero, $t\ (n = 45) = 2.078$, $p = 0.0218$). This is referred to as a "subsequent memory effect," or SME. (**D**) Positive Spearman correlation between the complexity of the assembly, a metric of how many neurons meaningfully influence assembly activity, and the predictive accuracy of increased assembly activation rate ($n = 45$, $r = 0.6129$, $p = 1.191e-5$). (**E**) Comparison of the observed t-statistic obtained by compare the distribution shown in (C) to zero to 1,000 t-statistics obtained after shuffling SE and UE event labels ($p = 0.0009$). (**F**) Comparison of the observed Spearman correlation coefficient to a null distribution obtained by shuffling UE and SE event labels ($p = 0.0009$). (**G**) Three example assemblies illustrating the relationship between assembly complexity and memory sensitivity (SME) (Wilcoxon two-group rank sum; left: $z\ (n = 18+87) = 1.9160$, $p = 0.0277$; middle: $z\ (n = 42+198) = 0.4783$, $p = 0.316$; right: $z\ (n = 81+159) = -1.477$, $p = 0.9301$). Schematics are as described in (A). Bar graphs are as described in (B). The SME z-score and complexity values for each example displayed are given by the correspondingly colored data point in (D). n.s. = not significant. † = $p < 0.10$. *$p < 0.05$. ***$p < 0.001$. Source data are provided as a source data file.

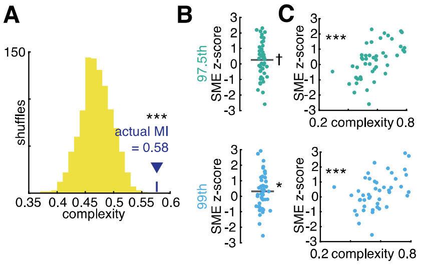

Supplementary Fig. 4. Control analysis for the relationship between assembly complexity and successful memory encoding. (**A**) Comparison of the average complexity value across assemblies to a null distribution obtained by shuffling the session-wide spike trains of all recorded neurons before re-isolating assemblies and calculating (permutation test, n = 1000 shuffles, p = 0.0009). (**B**) Distribution of SME z-scores using different thresholds for assembly activation (one sample t-test versus zero; top/teal: 97.5th percentile of observations, t(n = 45) = 1.489, p = 0.0718, bottom/cyan: 99th percentile of observation, t(n = 45) = 1.828, p = 0.0372). (**C**) Relationship between assembly memory sensitivity and complexity using assembly activation criteria outlined in (B) (Spearman rank correlation; top/teal: 97.5th percentile of observations, n = 45, r = 0.6369, p = 4.476e-6, bottom/cyan: 99th percentile of observation, n = 45, r = 0.4780, p = 0.0010). Source data are provided as a source data file.

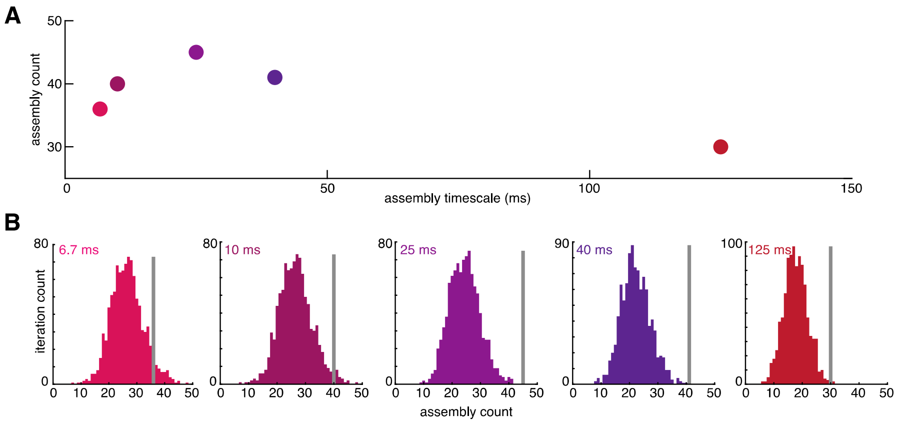

Supplementary Fig. 5. Selection of assembly timescale. (A) Number of assemblies identified at various timescales within gamma and theta frequency bands. (B) Comparison between the number of assemblies identified in the real data (line) and across 1,000 shuffles (distribution) at each investigated timescale, demonstrating a peak in absolute number and significance at 25 ms.

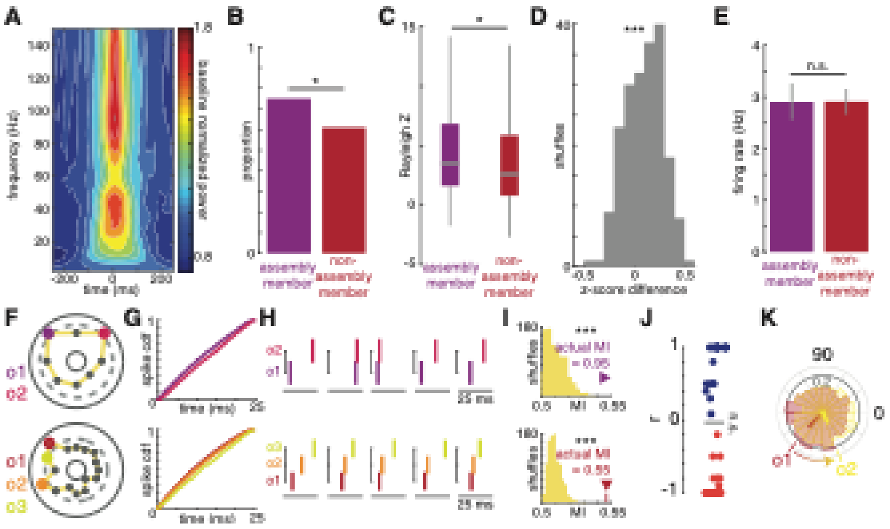

Supplementary Fig. 6. Gamma phase locking and assembly sequence development. (**A**) Time-frequency plot of the average baseline normalized (local baseline = 1) oscillatory power increases surrounding assembly activation events, demonstrating two peaks centered at 40 and 100 Hz. (**B**) Proportions of assembly member (purple) and non-member (red) neurons significantly phase locked to the underlying 40 Hz oscillation during memory behavior. Assembly members are more frequently phase locked to 40 Hz ($\chi^2(1) = 5.25$, $p = 0.022$). (**C**) Box plots demonstrating the distribution of z-scores obtained by performing a Rayleigh test on the spike phase distributions of all assembly member (purple) and non-member (red) distribution. Assembly members demonstrate stronger phase locking (z (305) = 1.74, p = 0.041). (**D**) Analysis controlling for the effect of firing rate on the strength of phase locking. The phase distributions of all neurons were randomly downsampled such that all neurons had the same number of spikes contributing to the Rayleigh test. Positive values indicated that the median Rayleigh z-score of assembly member neurons exceed that of non-members (one sample t-test, t (199) = 7.24, p = 4.787e-12). (**E**) Comparison of the baseline firing rates of assembly member and non-members (Wilcoxon two-group rank sum, z (305) = 0.26, p = 0.80). (**F**)-(**I**) Two additional examples of assemblies with member neurons colored according to the observed firing order within activation events. (F)-(I) follow the same convention as Fig. 2A-D. (**J**) Distribution of Spearman correlations between assembly neuron firing rate and assembly neuron firing order demonstrating no relationship. Each data point represents the correlation for one assembly. If an assembly had more than one possible sequence that maximized MI (n = 1), we took the average of correlation across all possible sequences. (**K**) Additional example of the relationship between firing order and preferred phase of 40 Hz phase locking for assembly member neurons. The plot follows the description for Fig. 3C. n.s. = not significant. *p < 0.05. ***p < 0.001. Source data are provided as a source data file.

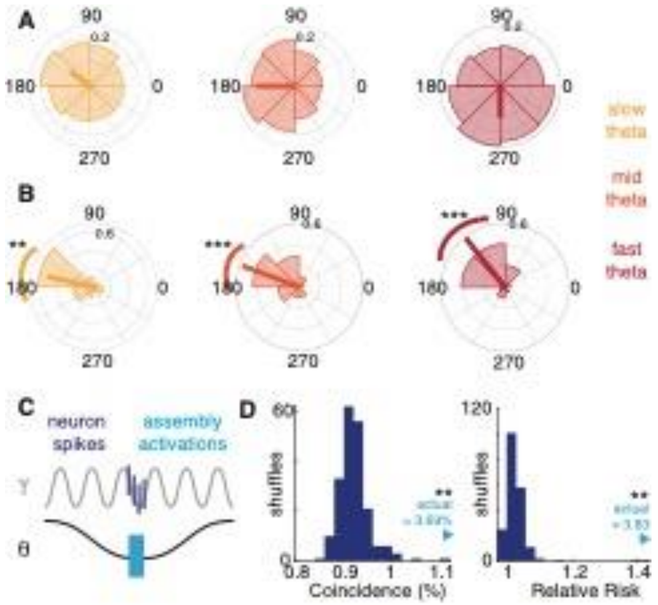

Supplementary Fig. 7. Theta phase locking assemblies and coincident ripple-like activity. (**A**) Phase histogram of assemblies demonstrating significant phase locking (FDR correction across channel and frequency, Q = 0.05) for slow (3 Hz, left), mid (5.5 Hz, middle), and fast (8 Hz, right) theta. (**B**) Histogram of mean phases for all assemblies demonstrating significant phase locking at each theta frequency tested. Note the proximity of the theta trough to the average preferred locking phase. The 95% confidence interval for mean phase is marked along the periphery of the phase histograms. All distributions were significantly non-uniform by the Rayleigh test—slow: $z(22) = 6.3$, $p = 0.0014$, 95% confidence interval (CI): 135-199 degrees, mid: $z(25) = 8.5$, $p < 0.001$, 95% CI: 134-186 degrees, fast: $z(24) = 8.7$, $p < 0.001$, 95% CI: 103-154 degrees. (**C**) Schematic demonstrating that assembly member neurons lock to gamma oscillations with assembly activations themselves tend to occur at the trough of underlying theta oscillations. (**D**) Shuffle histogram for the ripple-assembly activation coincidence rate (left, permutation test, $p = 0.005$,) and relative risk of assembly activations during and outside of ripple activity ($p = 0.005$, shuffle test, right). **$p < 0.01$. ***$p < 0.001$. Source data are provided as a source data file.

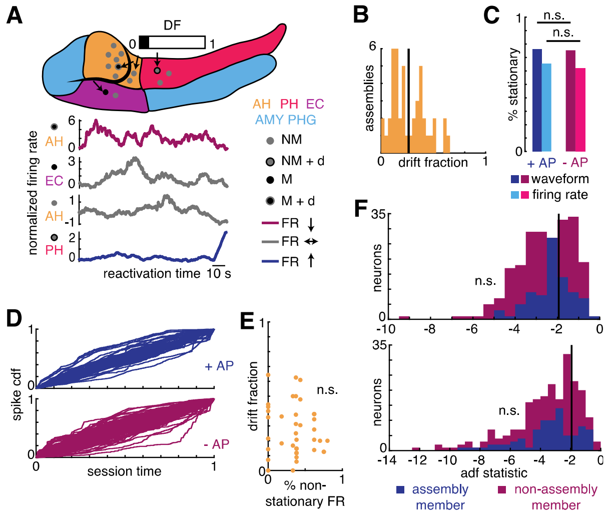

Supplementary Fig. 8. Signal non-stationarity does not explain assembly identification or the mnemonic relevance of assembly drift. (**A**) An additional example of assembly drift, following the same convention as described in Fig. 4A. (**B**) Histogram of DFs observed across assemblies. The line represents the overall drift fraction across all assemblies. (**C**) Comparison of the fractions of assembly member (blue) and non-member (red) neurons that are significantly stationary by the augmented Dickey-Fuller test. The populations did not significantly differ in the proportion of stable units by spike waveform (chi square test, $\chi^2(1) = 0.02$, $p = 0.88$) or firing rate ($\chi^2(1) = 0.34$, $p = 0.56$) criteria. (**D**) Cumulative distribution functions for the spiking activity of all assembly member (blue, top) and non-member (red, bottom) neurons across the recording session. (**E**) Demonstration of the lack of a correlation between assembly drift fraction and the number of non-stationary units by firing rate criteria isolated in the corresponding session (Spearman rank correlation, n = 45, r = -0.06, p = 0.69). Thus, non-stationarity in neuron firing rate is unlikely to explain the drift in neuronal contribution to assemblies across the experiment. (**F**) Histograms of augmented Dickey-Fuller statistics for assembly member (blue) and non-member (red) neurons using firing rate (top) and spike waveform criteria. The critical value above which the unit is not considered significantly stationary is displayed as a vertical black line. The distributions of test statistics do not differ for waveform (two-group rank sum; bottom, z (n = 84+223) = -0.0599, p = 0.9523) or firing rate (two-group rank sum; top z (n = 84+223) = 1.688, p = 0.0914) criteria. Thus, recording instability is unlikely to explain assembly identification. n.s. = not significant. Source data are provided as a source data file.

Supplementary Table 1. Participant characteristics. These data represent a subset of the participant population from a previous study[18]. We recorded 15 total sessions from these 13 participants, identifying 45 assemblies. * Indicates that this subject contributed 2 recording sessions with all others contributing a single session.

#	Sex	Age	Handed-ness	Epileptogenic zone	Epilepsy duration (y)
1	F	34	R	L temporal	15
2*	F	44	R	L medial temporal and R temporal	38
3	M	45	R	B temporal	15
4	M	20	R	R posterior temporal	10
5	F	52	R	R anterior temporal	11
6	M	31	L	R multifocal (poorly localized)	17
7	F	44	R	L anterior temporal and hippocampus	3
8	M	47	R	R frontal	31
9*	F	24	L	L temporal	12
10	F	25	R	-	-
11	M	29	A	R amygdala and hippocampus	10
12	M	22	R	R temporal, L hippocampus	3
13	F	35	R	L hippocampus, lingual gyrus, precuneus	5
